# Adipose‐derived mesenchymal stem cells induced PAX8 promotes ovarian cancer cell growth by stabilizing TAZ protein

**DOI:** 10.1111/jcmm.16511

**Published:** 2021-04-08

**Authors:** Yijing Chu, Chengzhan Zhu, Qianqian Wang, Meixin Liu, Wei Wan, Jun Zhou, Rendong Han, Jing Yang, Wenqiang Luo, Chong Liu, Huansheng Zhou, Min Li, Fengsheng Yu, Yuanhua Ye

**Affiliations:** ^1^ Department of Obstetrics The Affiliated Hospital of Qingdao University Qingdao China; ^2^ Department of Hepatobiliary and Pancreatic Surgery The Affiliated Hospital of Qingdao University Qingdao China

**Keywords:** adipose‐derived mesenchymal stem cells, ovarian cancer, PAX8, protein stability, TAZ

## Abstract

Our previous studies have shown that the Adipose‐derived mesenchymal stem cells (ADSCs) can regulate metastasis and development of ovarian cancer. However, its specific mechanism has yet to be fully revealed. In this study, an RNA‐seq approach was adopted to compare the differences in mRNA levels in ovarian cancer cells being given or not given ADSCs. The mRNA level of paired box 8 (PAX8) changed significantly and was confirmed as an important factor in tumour‐inducing effect of ADSCs. In comparison with the ovarian cancer cells cultured in the common growth medium, those cultured in the medium supplemented with ADSCs showed a significant increase of the PAX8 level. Moreover, the cancer cell growth could be restricted, even in the ADSC‐treated group (*P* < .05), by inhibiting PAX8. In addition, an overexpression of PAX8 could elevate the proliferation of ovarian cancer cells. Moreover, Co‐IP assays in ovarian cancer cells revealed that an interaction existed between endogenous PAX8 and TAZ. And the PAX8 levels regulated the degradation of TAZ. The bioluminescence images captured in vivo manifested that the proliferation and the PAX8 expression level in ovarian cancers increased in the ADMSC‐treated group, and the effect of ADSCs in promoting tumours was weakened through inhibiting PAX8. Our findings indicate that the PAX8 expression increment could contribute a role in promoting the ADSC‐induced ovarian cancer cell proliferation through TAZ stability regulation.

## 1 INTRODUCTION

The epithelial ovarian cancer (EOC) remains one of primary causes of mortality in gynaecologic cancer patients, partially because of the fact that although standard surgeries or platinum/paclitaxel‐based chemotherapy is performed, ultimately chemoresistant disease will redevelop in most patients.^1^, ^2^, ^3^ Ovarian cancer is a distinctive solid tumour type that originates, metastasizes and reoccurs at the same region‐the abdominal cavity, which forms its specific microenvironment.^4^ The tumour microenvironment (TME) is constituted by immunocytes, mesenchymal stem cells and non‐cellular components between the tumour tissue.^5^ Recent studies have shown that TME contributes significantly to the metastasis and drug resistance of ovarian cancer.^6^ As revealed in our prior study, the growth rate and invasion of ovarian cancer could be enhanced dramatically by applying ADSCs in the omentum, suggesting a further progression of ovarian cancer.^6^, ^7^, ^9^ Furthermore, the TME that can induce omental metastasis in the omentum develops in this process^10^; however, its specific mechanism is not fully investigated.

PAX8 is one gene member among the paired box gene family. It encodes proteins that contain an evolutionarily conserved paired box domain.^11^ PAX8 is crucial in dictating the cell fate during the development of organs and tissues; in mature normal tissues, its expression is present in kidney, thyroid and Fallopian tubes, but not in the ovarian epithelium.^12^ The staining results reveal a significant increase of PAX8 expression in the tissues of ovarian and endometrial cancer compared to the adjacent tissues, especially in serous tumours such as ovarian cancer and clear cell carcinoma.^13^, ^14^ Moreover, patients with ovarian cancer that have high PAX8 levels are more likely to relapse and usually show a poor clinical prognosis.^15^

The Hippo pathway is an evolutionarily conserved signalling cascade regulating several physiological functions, including cell growth and survival, mobility, regeneration and differentiation.^16^ The Hippo pathway can be activated by both intrinsic and extrinsic signals.^17^ The core of the Hippo pathway contains a kinase cascade, MST1/2 and LATS1/2, and downstream effectors, transcriptional coactivators YAP/TAZ.^18^ TAZ (transcriptional coactivator with PDZ‐binding motif, also known as WWTR1) is a transcriptional coactivator in regulating the function of several transcription factors and is largely involved in the modulation of cell proliferation/differentiation, tissue development and organ morphogenesis.^19^ It has been demonstrated in studies that the transcription factor activity can be regulated by interacting with other proteins, such as coactivators and co‐repressors; and as a coactivator, TAZ can interact with PAX8.^20^

In our previous studies, we found omental ADSCs advanced ovarian cancer cell proliferation and invasion, but its specific mechanism remained unknown. The RNA‐seq analysis was performed in this study to investigate the changes in ovarian cancer cells treated with ADSCs conditional medium. It was found that PAX8 was up‐regulated significantly in ovarian cancer cells treated with ADSC, accompanied by the hippo signalling pathway activation. This study aims to study the effect of PAX8 induced by ADSCs on ovarian cancer's growth and invasion through stabilizing TAZ.

## 2 MATERIALS AND METHODS

### 2.1 Cell culturing

The cell lines of human ovarian cancer cells, SKOV3, A2780, ES2 and HO8910 were procured from the Type Culture Collection China Centre. The DMEM/F12 (Gibco) containing 10% foetal bovine serum and 1% penicillin/streptomycin was applied to culture all three ovarian cancer cell lines. Cells were kept in an incubator at a temperature of 37°C at 5% CO_2_.

### 2.2 Conditioned medium preparation and ADSCs identification

Conditioned medium preparation and ADSCs identification were performed as previously described.^8^ In brief, ADSCs were grown to 80% confluence and discarded the medium. Then, they were cultured in serum‐free DMEM/F12 for 24 hours, and the medium was collected, centrifuged and filtered through 0.22‐µm filters (Millipore).

### 2.3 RNA‐seq

SKOV‐3 cells treated with or without ADSC CM were stored at −80°C after being isolated in 1 mL TRIzol reagent (Thermo Fisher Scientific). The RNA‐seq libraries were prepared as per the protocol of the Illumina TruSeq RNA Sample Prep Kit. And the sequencing process was conducted on a MiSeq device. Relevant experiments were conducted at the Annoroad Gene Technology. And the data were analysed through RSEM software. All RNA‐seq data could be obtained in the Sequence Read Archive (SRA) under accession nos. SRR11492552‐SRR11492557.

### 2.4 Transient transfection and lentivirus infection

Two cell lines of ovarian cancer cells were cultured for 24 hours in 10‐cm plates until the status turned subconfluent. siRNAs Transient transfections and lentivirus infection were performed with Lipofectamine 2000 (Invitrogen). The SKOV3 cell line was transfected with lentivirus containing a PAX8‐specific shRNA or a non‐targeting scrambled shRNA (Hanbio). The A2780 cell line was transfected with control lentivirus or lentiviral constructs expressing full‐length PAX8 (Hanbio).

The transfected cells were treated with puromycin to isolate single clones to establish cell lines with stable down‐regulation or up‐regulation of PAX8.

The sequences of the siRNAs and shRNAs used are as following: siNC, 5′‐GCAAGCTGACCCTGAAGTT‐3′; siRNA1, 5′‐GCCAGAACCCTACCATGTT‐3′; siRNA2, 5′‐CCAGAACCCTACCATGTTT‐3′; shNC, 5′‐GCAAGCTGACCCTGAAGTT‐3′; shRNA, 5′‐GCCAGAACCCTACCATGTT‐3′.

### 2.5 Quantitative real‐time PCR (qRT‐PCR)

Total RNA was extracted by a TRIzol reagent (Takara). Reverse transcription kits (Invitrogen) were subsequently used for synthesis of complementary DNAs. qRT‐PCR was conducted by using SYBR Premix Ex Taq (Takara) and an ABI 7500 Sequencing Detection System. GAPDH was utilized to serve as a control.

The following primer sequences were used: PAX8, forward, 5′‐GATCCTCACTCACCCTTCGC‐3′, reverse, 5′‐TAACCACACAGGGAGTGTGC‐3′; TAZ, forward, 5′‐CTCACATCCTGGCGACTCTC‐3′, reverse, 5′‐CACGAGCTAGGCTTCGGATT‐3′; GAPDH forward, 5′‐AATGGGCAGCCGTTAGGAAA‐3′, reverse, 5′‐GCGCCCAATACGACCAAATC‐3′.

### 2.6 Western blotting

Cells were lysed in RIPA buffer (Sigma‐Aldrich) with the protection of ice. The cell lysate was centrifuged at 12 000 *g* and was treated with LDS sample buffer. After that, protein aggregates were separated with SDS‐PAGE gels and electrotransferred onto the polyvinylidene fluoride (PVDF) membrane (Bio‐Rad). The PVDF membranes were then blocked using 5% instant skim milk and subjected to incubation with primary antibodies at 4°C overnight, which is followed by being incubating with secondary antibodies (1:1000; CST). The protein‐antibody complexes were detected and quantified using a chemiluminescence detection system (Bio‐Rad).

The primary antibodies used in Western blotting were as follows: PAX8 (ab53490, Abcam; 1:1000), TAZ (ab205270, Abcam; 1:1000), YAP (ab52771, Abcam; 1:1000) and GAPDH (ab181603, Abcam; 1:5000).

### 2.7 Cell proliferation analysis

Cells (5 × 10^3^/well) were seeded in the 96‐well plates. A CCK‐8 reagent (Thermo Fisher Scientific) was used daily to detect the proliferation rate of ovarian cancer cells. Each well was added with the CCK‐8 reagent and cultured for an extra 1.5 hours. After that, colorimetric assays were conducted to measure the OD value of each well at a wavelength of 450 nm by using a microplate reader. Three separate experiments were performed to determine the growth curves.

### 2.8 Colony formation assay

A total of 500‐1000 ovarian cancer cells were incubated in 6‐well plates for 14 days. Paraformaldehyde was then used to fix the colonies which were afterwards stained with a crystal violet solution for visualization.

### 2.9 Luciferase reporter assays

Cells (5 × 10^3^/well) were seeded in 96‐well plates. The synthetic TEAD luciferase reporter (Addgene), and Renilla reporter constructs (Promega) were co‐transfected into stably infected cells with Lipofectamine 3000 (Thermo Fisher Scientific). After 24 hours, Dual‐Luciferase Reporter Assay Kit (Promega) was used to measure luciferase activity. The Renilla activity was used to normalize the luciferase reporter activity.

### 2.10 Co‐immunoprecipitation (Co‐IP)

Preparation for cell lysates was conducted using a lysis buffer (Pierce). The lysates were then centrifuged, and the supernatant was cleared by being incubated with Protein A/G magnetic beads (Thermo Fisher Scientific) at 4°C for 1 hour. The pre‐cleared supernatant was immunoprecipitated using the primary antibodies TAZ (ab242313, Abcam; 1:1000) at 4°C overnight. The protein complexes were then incubated with Protein A/G magnetic beads at 4°C for 1 hour and were then subjected to Western blot analysis.

### 2.11 Orthotopic ovarian cancer modelling

The orthotopic mouse ovarian cancer model was established using an in vivo imaging system. The behaviour of tumour cells was then closely monitored as previous described.^8^ In brief, the ovarian cancer cell line expressing firefly luciferase (SKOV3‐Luc) was created after the SKOV3 cell line was transfected with lentiviral particles (GenePharma Co.) and cells with high luciferase expression were screened out using puromycin. The SKOV3‐Luc cells were harvested and then preserved in PBS at a ratio of 10 000 cells/μL on ice. We then anaesthetized and performed surgery on the mice. Under a microscope, the mouse ovarian fat pad was excised. Then, the ovary was fixed and a needle was inserted into the bursa. A total of 5 μL cell suspension was gently injected into the tissue between the cyst and ovary. The mouse reproductive tract was then gently replaced and sutured. The recovering mice were monitored and provided with a heat source. The thirty female BALB/c‐nu nude mice that underwent the above‐described surgery were randomly divided into the following four groups: shPAX8 + CM, shPAX8 + PBS, shNC + CM and shNC + PBS. ADSC CM (500 µL) or PBS (500 µL) was injected intraperitoneally on day 7 after surgery.

Before the mice were anaesthetized, they were injected intraperitoneally with D‐luciferin, a firefly luciferase substrate. Imaging studies were conducted on all mice weekly, for 4 consecutive weeks. The bioluminescence intensity of tumours was detected with Living Image software (Lumina II Living Image version 4.3). The total signal counts were normalized to the image acquisition time to count the whole area of tumours.

### 2.12 Statistical analysis

Statistical analysis was performed with unpaired two‐tailed Student's *t* test, and all data are presented as mean ± standard error mean (SEM) deviations from more than three separate measurements. GraphPad Prism (GraphPad) was adopted for data analysis. The statistical significance was set at *P* value < .05.

## 3 RESULTS

### 3.1 Global transcriptome characterization of ovarian cancer cells regulated by ADMSCs and up‐regulation of PAX8 in ovarian cancer tissues

**FIGURE 1:**
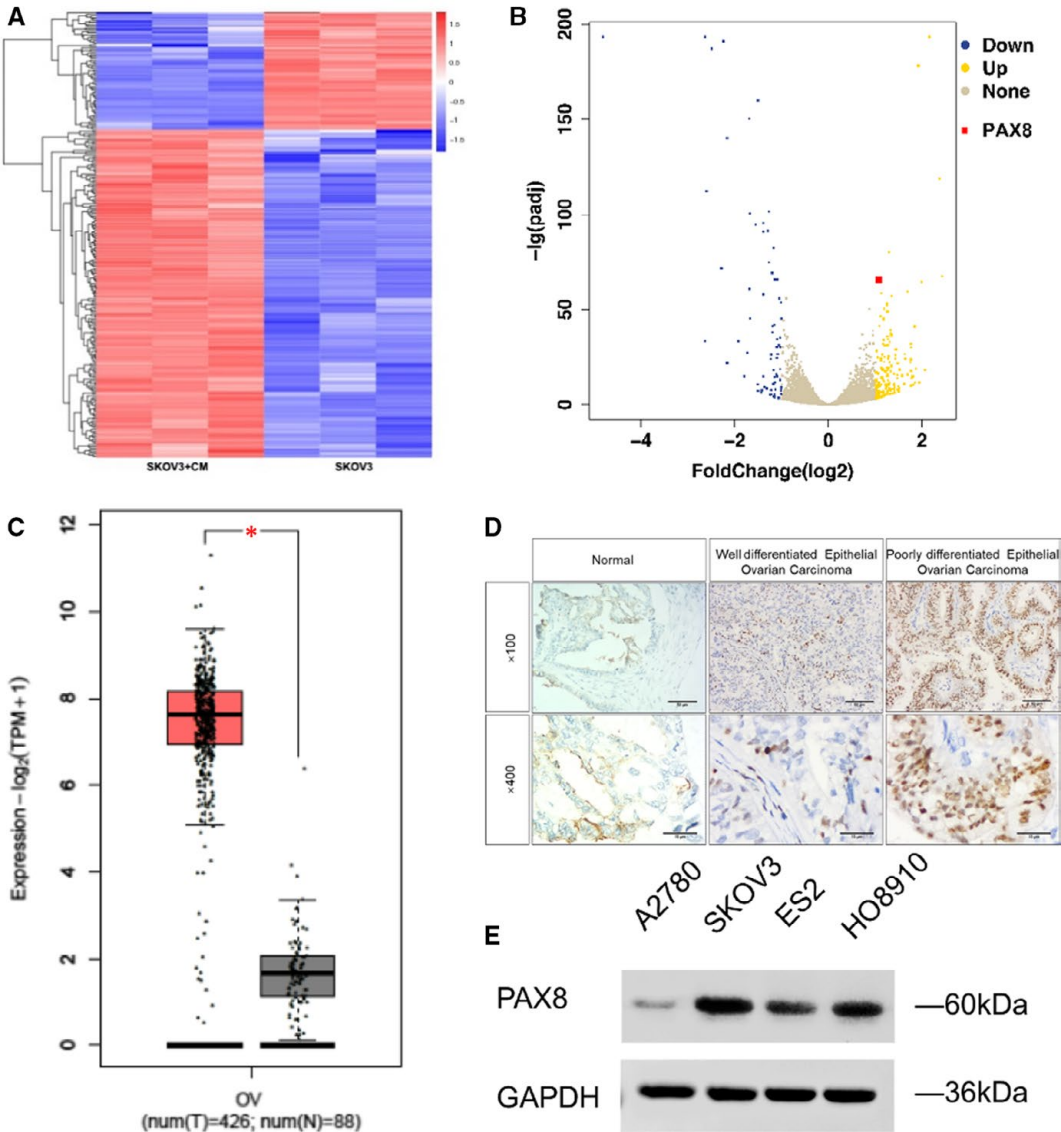
PAX8 is overexpressed in ovarian cancer. A, Heat map of differential mRNA expression between SKOV3 + PBS and SKOV3 + ADSCs CM. B, Volcano map of differential mRNA expression between SKOV3 + PBS and SKOV3 + ADSCs CM. C, PAX8 gene expression in ovarian cancer compared with that in normal tissues. Data were extracted from the GEPIA database (http://gepia2.cancer‐pku.cn/). D, PAX8 gene expression in ovarian cancer tissue and in normal ovarian tissues was examined IHC. E, Western blot analysis for PAX8 levels in A2780, SKOV3, ES2 and HO8910 ovarian cancer cell lines. GAPDH was used for normalization. Student's *t* test: *P < .05

To better understand the changes in ovarian cancer cells that occurred after being treated with ADSC, we compared the transcriptomes of SKOV3 cells cultured for 48 hours with or without ADSC CM. By RNA‐seq analysis, we found 195 up‐regulated and 70 down‐regulated genes, showing more than twofold change (Figure 1A). Top 10 up‐regulated genes were listed, as in Table 1, including PAX8. We analysed all these 10 genes by literature retrieval, and many studies revealed that PAX8 was highly expressed in cancers and play a role in cancer progression.^21^ Moreover, PAX8 was found to be highly expressed in ovarian cancer and has exhibited pro‐proliferative effect.^22^, ^23^, ^24^ In addition, Chai et al^25^ reported that higher PAX8 expressed patients with epithelial ovarian cancer exhibited shorter post‐operative survival times. Then, the PAX8 expressions in ovarian cancer tissues and fallopian tubes tissue (normal) were compared, revealing a much higher level of PAX8 expression in ovarian cancer tissues (Figure 1B,C). In addition, the PAX8 expressions in 4 cell lines of ovarian cancer were detected by Western blot, and the results showed that SKOV3 cells expressed a higher level of PAX8 while A2780 cells expressed a relatively lower level (Figure 1D). In summary, our analyses reveal that PAX8 is closely associated with ovarian cancer and may involve in the changes of ovarian cancer cells mediated by ADSCs.

**TABLE 1:** Top 10 increased expressed protein in SKOV‐3 cells treated with ADSCs CM

Accession	Description	Gene name	Ratio	*P* value
Q96BD0	Solute carrier organic anion transporter family member 4A1	SLCO4A1	4.818723616	3.51E‐194
P15121	Aldo‐keto reductase family 1 member B	AKR1B1	4.052241051	5.22E‐179
P09341	C‐X‐C motif chemokine ligand 1	CXCL1	5.800582242	2.17E‐119
P07305	H1.0 linker histone	H1F0	2.60659945	5.59E‐81
Q9BTP6	Zinc finger BED‐type containing 2	ZBED2	6.494545455	3.46E‐68
Q06710	Paired box 8	PAX8	2.15660156	2.34E‐65
Q96DY2	IQ motif containing D	IQCD	4.491027732	2.58E‐65
Q8NFP4	MAM domain‐containing glycosylphosphatidylinositol anchor 1	MDGA1	3.560585885	4.07E‐60
Q8IZV5	Retinol dehydrogenase 10	RDH10	2.32006466	2.84E‐59
P24821	Tenascin C	TNC	2.73256262	7.93E‐58

### 3.2 Higher expression of PAX8 was induced by ADSCs in ovarian cancer cells

**FIGURE 2:**
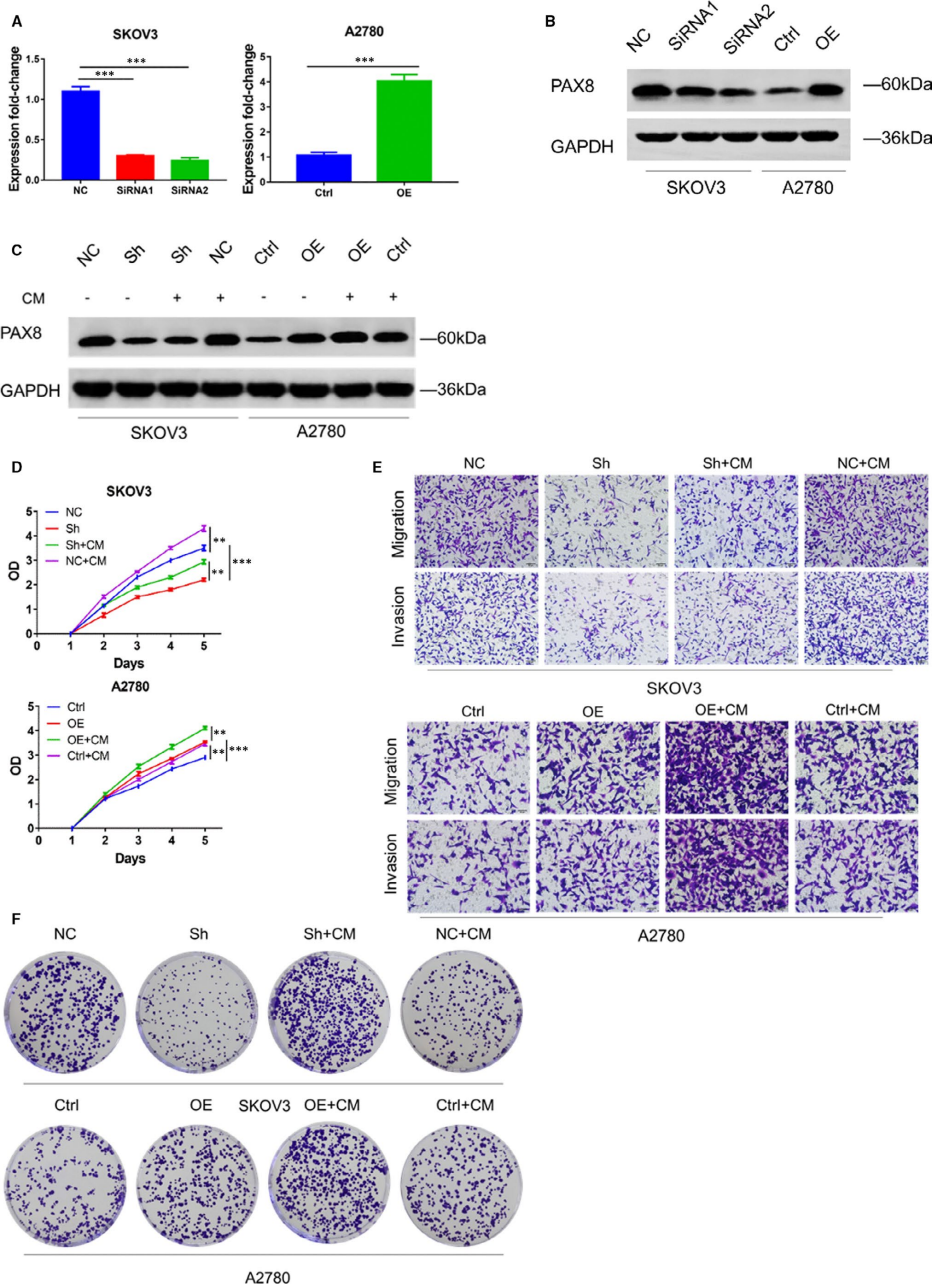
ADMSCs promote the proliferation of ovarian cancer cells in vitro via PAX8. A, qRT‐PCR analysis and (B) Western blotting analysis of PAX8 protein levels in SKOV3 cells transfected with siRNAs and A2780 cells transfected with Ctrl or PAX8‐expressing lentivirus. Assays were performed in triplicate. C, Western blotting analysis of PAX8 protein levels in stably transfected ovarian cancer cells cultured alone or with ADSC CMs. D, OD values from CCK‐8 assays of indicated groups to evaluate cell proliferation. E, Comparison of ovarian cancer cells invasion in indicated groups using Transwell assays. F, The cloning efficiency of ovarian cancer cells were evaluated by Colony formation assay. The assays were performed in triplicate. Student's *t* test: ***P < .005, **P < .01

First, through the qRT‐PCR and Western blot analysis, we confirmed that PAX8 expression could be inhibited by the PAX8 siRNA and elevated by overexpression vector in SKOV3 and A2780 cells (Figure 2A,B). Then, the effect of ADSCs on PAX8 level was studied in ovarian cancer cells. Western blot was adopted to evaluate the PAX8 protein level in the siRNA‐silenced SKOV3 cells and in overexpression vector‐treated A2780 cells with or without ADSC CM (Figure 2C). The results confirmed that compared with cells cultured in the normal medium, PAX8 was expressed at much higher levels in the two ovarian cancer cell lines treated with ADSC CM. Despite the presence of ADSC CM, the PAX8 siRNA suppressed PAX8 expression in SKOV3 cells, and the plasmids elevated PAX8 expression in A2780 cells. Based on these data, it can be confirmed that ADSCs induce an overexpression of PAX8 in ovarian cancer cells.

### 3.3 ADSCs promoted ovarian cancer cell proliferation by up‐regulating PAX8 expression

After confirmation of ADSCs causing PAX8 overexpression in ovarian cancer cells, the role of PAX8 in regulating cancer cell proliferation was investigated next. ADSC CM was utilized for treating the ovarian cancer cells transfected by the shRNA and plasmids, and the proliferation rate was detected with a CCK‐8 assay. Although present with ADSC CM, inhibiting PAX8 also restrained the proliferation of cancer cells, and the PAX8 overexpression had the similar proliferation‐promoting effect of ADSCs CM (Figure 2D). Consistent with the result of CCK‐8 assays, the colony formation assays showed that ADSCs CM contributed to increases of both colony sizes and cell numbers in two ovarian cancer cell lines. Those increases were decreased in shRNA‐transfected SKOV3 cells, and the overexpression plasmids had a similar effect as ADSCs CM in A2780 cells (Figure 2E). Taken together, ADSCs may advance the proliferation of ovarian cancer cells through the PAX8 up‐regulation.

### 3.4 PAX8 increases TAZ expression by stabilizing TAZ proteins

**FIGURE 3:**
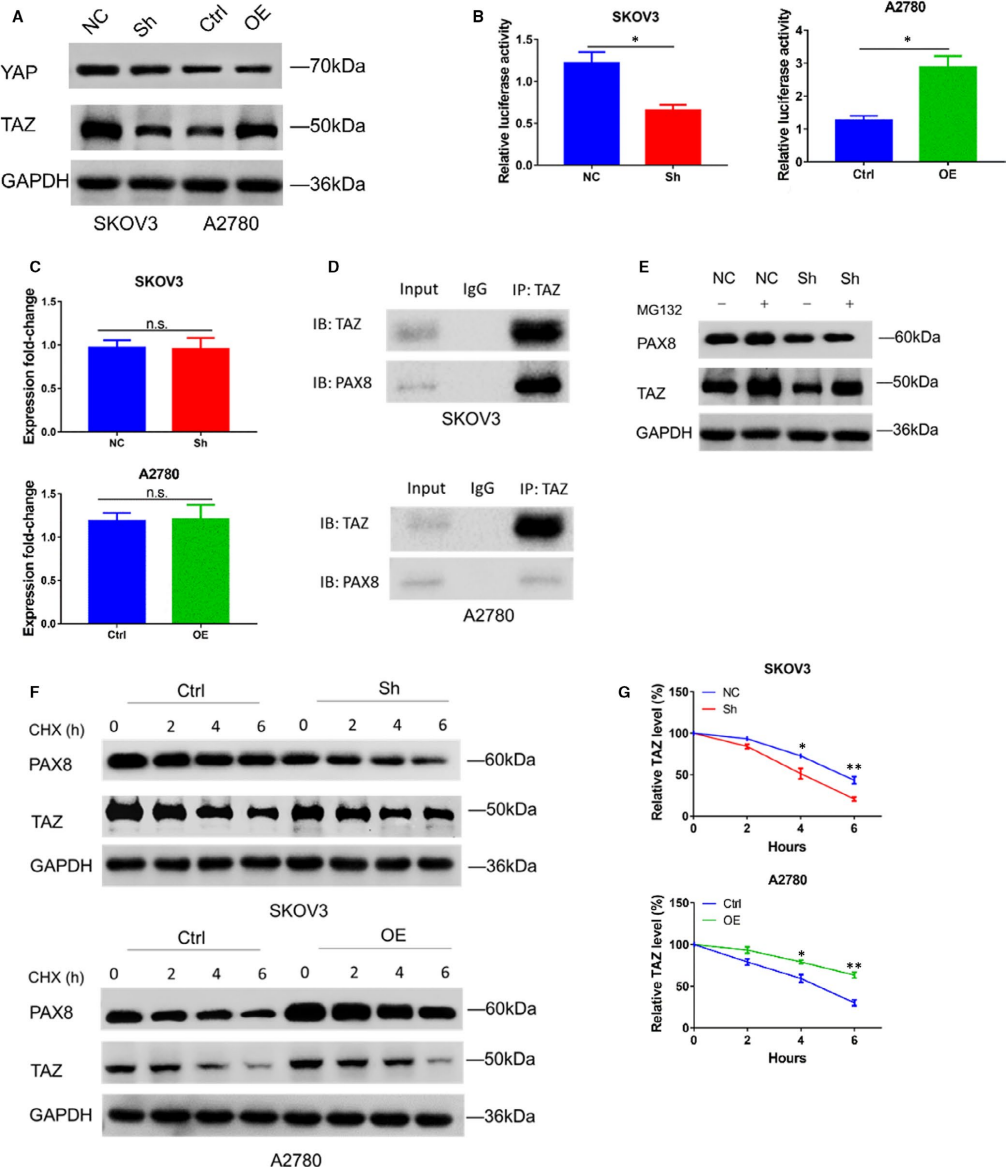
PAX8 is required for TAZ protein stability. A, Western blotting analysis of YAP and TAZ from stably transfected cells. B, Relative TEAD reporter activity in stably transfected SKOV3 and A2780 cells. C, RT‐PCR results performed using total RNA from the indicated cell lines. GAPDH mRNA was amplified as control. D, Co‐IP results from SKOV3 and A2780 showing PAX8 coprecipitates with TAZ. E, Representative Western blot of TAZ expression at baseline or after incubation with MG132 (25 μmol/L) treatment for 8 h. GAPDH was used for normalization. F, Western blot analysis of TAZ proteins in indicated cells treated with CHX (30 μg/mL) for 0, 2, 4 or 6 h. G, CHX release profiles. Data are represented as the mean ± SEM. Student's *t* test: **P < .01, *P < .05, n.s., not significant

TAZ is reported to act as a transcriptional coactivator of PAX8 to regulate its expression in the thyroid (TAZ function as a coactivator of both Pax8 and TTF‐1, two transcription factors affecting thyroid differentiation) and is the homologous gene of YAP (YAP and TAZ are the downstream core elements of classical hippo pathway).^20^, ^26^ We wonder if it is possible that PAX8 is involved in TAZ‐mediated transcription activity. To verify this, we examined the expression of two coactivators, TAZ and its paralog YAP, in the Hippo signalling pathway. We established the PAX8 knock‐down SKOV3 cell line and PAX8 over expression A2780 cell line, and Western blot analysis revealed that the TAZ protein level was reduced in SKOV3‐sh cells but increased in A2780‐OE cells (Figure 3A). In addition, we also confirmed the effect of PAX8 on TAZ‐mediated transcription activity using a TEAD‐dependent reporter (Figure 3B). Having previously established that PAX8 is necessary for RB protein stability (PAX8 can regulate E2F1 transcription and stabilize RB protein to promote tumour cell proliferation),^27^ we hypothesized that PAX8 could also regulate TAZ in protein level. First, we ruled out the possibility that PAX8 regulates TAZ transcription. The qRT‐PCR results showed no substantial changes in TAZ transcript levels following changes of PAX8 levels (Figure 3C). Then, we performed Co‐IPs in both SKOV3 and A2780 cell lines. PAX8 was found in complexes with antibodies against TAZ in both cell lines (Figure 3D), indicating an interaction between the endogenous PAX8 and TAZ in SKOV3 and A2780 cells. Further experiments showed that the treatment of proteasome inhibitor (MG132) caused an accumulation of TAZ in PAX8‐depleted cells (Figure 3E). The cycloheximide (CHX), a protein synthesis inhibitor, was also used. Western blot was performed in presence of CHX after 6 hours and showed that the loss of PAX8 accelerated TAZ degradation in SKOV3 cells while overexpression of PAX8 slowed TAZ degradation in A2780 cells (half‐life, ~6 hours vs ~4 hours, SKOV3‐NC and SKOV3‐Sh; ~4 hours vs >6 hours, A2780‐Ctrl and A2780‐OE; respectively; Figure 3F,G). Collectively, PAX8 might be associated with TAZ protein stability and could increase TAZ expression in ovarian cancer cell lines.

### 3.5 ADSCs advanced the growth and metastasis of ovarian cancer in vivo through PAX8 up‐regulation

**FIGURE 4:**
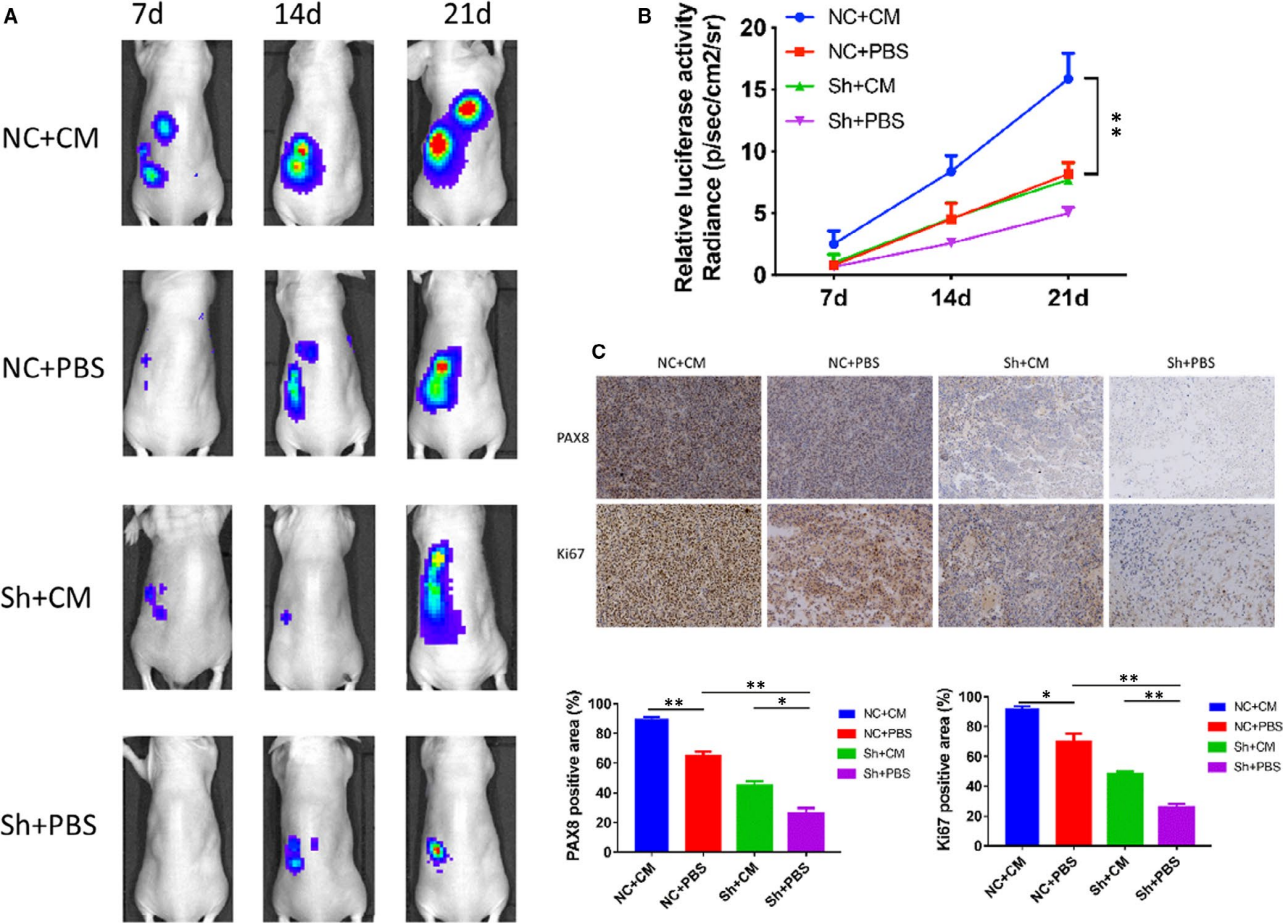
ADSCs promote the proliferation of ovarian cancer cells in vivo via PAX8. A, An in vivo imaging system was used to monitor the luminescence intensity of SKOV3 xenografts, which represented tumour growth and metastasis. B, Living Image software was used to analyse the tumour bioluminescence intensity weekly for 6 animals per group. Quantitative results for the normalized image counts are shown. C, Immunohistochemical staining for Ki67 and PAX8 in the orthotopic tumour xenografts is shown. Results of the quantitative analysis of Ki67‐ and PAX8‐positive areas in xenografts are presented in the right panel. *P < .05, **P < .01 and ***P < .001

A series of xenograft experiments were performed to investigate the effect of PAX8 on the capability of ADSCs to advance ovarian cancer development in vivo. Orthotopic mouse models were established successfully 7 days after surgeries. The bioluminescent signals detectable in the shNC + CM group were much stronger compared with the other groups, and the signals were significantly different in the four groups (Figure 4A,B, *P* < .001). In comparison with the other groups, the tumour growth‐promoting capability of ADSCs was significantly decreased in the shPAX8 group. The Ki67 expression in xenograft tumour tissues was analysed to study the proliferation rate of transplanted cells of ovarian cancer. The result showed high expression levels of Ki67 in ovarian tumour tissues of the shNC + CM group. We also detected higher levels of PAX8 expression in the shNC + CM group than in the other groups (Figure 4C). Therefore, the results show that ADSCs can advance the ovarian cancer growth rate and metastasis, and silencing PAX8 expression can reverse this effect.

## 4 DISCUSSION

Growing evidence has indicated that other types of cells coexisting in the tumour microenvironment also contribute important roles in the tumour development, including proliferation, angiogenesis, invasion and metastasis.^28^, ^29^ Therefore, the tumour microenvironment is a critical determining factor in the cancer pathogenesis and is characterized by complicated interactions between multipotent stem cells, fibroblasts, inflammatory/immune cells, cell factors and extracellular matrix.^30^ In usual conditions, these stromal components inhibit the carcinogenic process which is correlated with organismal survival; nevertheless, in a tumour‐associated neighbourhood, the stroma can be altered by various stimuli to advance tumour progression.^5^, ^31^ Our previous studies showed that in ovarian cancer cells, omental ADSCs could advance their growth and invasion and was involved in the omental pre‐metastatic niche formation in ovarian cancer.^7^ However, much remains unknown on the exact changes in ovarian cancer cells caused by ADSCs.

PAX8 is a lineage‐specific transcription factor involved in the epithelial development of the fallopian tube and express in high‐grade serous ovarian carcinoma derived from both the fallopian tube and ovarian surface epithelium.^21^, ^26^ With respect to other EOC histotypes, PAX8 is expressed at higher levels in clear cells and endometrioid tumours and at lower levels in mucinous tumours.^13^ An RNA‐seq analysis of ovarian cancer cells, given or not given ADSC CM, reveals PAX8 is among the top 10 differentially expressed genes. Subsequently, the role of ADSCs in advancing ovarian cancer development was explored, and the effect of PAX8 was also identified during this process. Our study showed that in comparison with the normal culture medium, the ADSC CM‐supplemented medium could significantly increase PAX8 levels in ovarian cancer cells. Furthermore, the capability of ADSCs to promote tumour growth, metathesis and invasion was inhibited by silencing PAX8 in ovarian cancer cells both in vitro and in vivo.

As one of mammalian orthologous Yorkie genes, transcriptional coactivator with a PDZ‐binding motif (TAZ) is a transcriptional coactivator of the Hippo pathway of Drosophila.^32^ Overexpression or hyperactivation of TAZ has been frequently seen in human cancers, where TAZ was considered as essential for the development and sustainability of neoplasia.^26^, ^33^, ^34^ TAZ is closely regulated as a transcriptional coactivator at certain layers.^35^ TAZ may be phosphorylated directly by LATS1/2 (the Hippo signalling) and degenerated further by the SCF/CRL1 (β‐TrCP) E3 ligase.^19^, ^26^, ^33^ The capability of TAZ for regulating protein stability is important for its involvement in the growth, apoptosis, migration and invasion of cells of multiple human cancers, as well as the epithelial‐mesenchymal transition (EMT) and stemness.^36^, ^37^, ^38^ Overall, TAZ contributes an critical role in carcinogenesis by regulating various respects of cancer cells, indicating that TAZ could function as a promising candidate for cancer diagnosis or therapy.^39^, ^40^ A better knowing of the molecular mechanisms of TAZ mediating tumour development would help developing TAZ‐targeted cancer therapies. Our results confirm that ADSCs are necessary for TAZ protein stability and also promote TAZ transcription in the ovarian cancer through PAX8 up‐regulation. Although the specific mechanisms of how ADSCs mediate the TAZ stability in ovarian cancer cells through PAX8 were still unclear, an association between ADSCs, PAX8 and TAZ in ovarian cancer sheds novel light on the mechanism of ADSCs advancing the ovarian cancer metastasis.

In sum, ADSCs increase the ovarian cancer cell proliferation at least partially by up‐regulating the PAX8 expression and by maintaining TAZ stability in ovarian cancer cells. PAX8 can potentially regulate cancer progression, that is ADSC‐induced tumour development, and participated in modulating the interaction between TME and primary tumours. Our study provides insights into developing ovarian cancer‐specific preventive therapy in the future.

## CONFLICTS OF INTEREST

The authors declare no potential conflicts of interest.

## AUTHOR CONTRIBUTIONS

**Yijing Chu:** Conceptualization (equal); Writing‐original draft (lead). **Chengzhan Zhu:** Formal analysis (equal); Writing‐review & editing (equal). **Qianqian Wang:** Data curation (equal); Resources (equal); Supervision (equal). **Meixin Liu:** Methodology (equal); Supervision (equal). **Wei Wan:** Investigation (equal); Resources (equal). **Jun Zhou:** Conceptualization (equal). **Rendong Han:** Formal analysis (equal). **Jing Yang:** Data curation (equal); Methodology (equal). **Wenqiang Luo:** Formal analysis (equal); Visualization (equal). **Chong Liu:** Data curation (equal); Formal analysis (equal). **Huansheng Zhou:** Data curation (equal); Formal analysis (equal). **Min Li:** Formal analysis (supporting); Writing‐original draft (supporting). **Fengsheng Yu:** Conceptualization (equal); Funding acquisition (equal); Writing‐review & editing (lead). **Yuanhua Ye:** Conceptualization (lead); Funding acquisition (lead); Resources (lead); Writing‐review & editing (supporting).

## Data Availability

The data that support the findings of this study are included within the article and openly available in Sequence Read Archive (SRA) labelled as no. SRR11492552‐SRR11492557.

## References

[1] Richardson DL . New and novel therapies for gynecologic cancers. Semin Oncol Nurs. 2019;35(2):217‐219. 10.1016/j.soncn.2019.02.009 30876683

[2] Siegel RL , Miller KD , Jemal A . Cancer statistics, 2018. CA Cancer J Clin 2018;68(1):7‐30. 10.3322/caac.21442 29313949

[3] Yan S , Frank D , Son J , et al. The potential of targeting ribosome biogenesis in high‐grade serous ovarian cancer. Int J Mol Sci. 2017;18(1):210. 10.3390/ijms18010210 PMC5297839 28117679

[4] Emmings E , Mullany S , Chang Z , Landen CN Jr , Linder S , Bazzaro M . Targeting mitochondria for treatment of chemoresistant ovarian cancer. Int J Mol sci. 2019;20(1):229. 10.3390/ijms20020229 PMC6337358 30626133

[5] Atiya H , Frisbie L , Pressimone C , Coffman L . Mesenchymal Stem Cells in the Tumor Microenvironment. Adv Exp Med Biol.. Chapter 3. 2020:1234 31‐42. 32040853 10.1007/978-3-030-37184-5_3

[6] Roma‐Rodrigues C , Mendes R , Baptista PV , Fernandes AR . Targeting tumor microenvironment for cancer therapy. Int J Mol Sci. 2019;20(4):840. 10.3390/ijms20040840 PMC6413095 30781344

[7] Chu Y , Tang H , Guo Y , et al. Adipose‐derived mesenchymal stem cells promote cell proliferation and invasion of epithelial ovarian cancer. Exp Cell Res. 2015;337(1):16‐27. 10.1016/j.yexcr.2015.07.020 26209607

[8] Chu Y , You M , Zhang J , et al. Adipose‐derived mesenchymal stem cells enhance ovarian cancer growth and metastasis by increasing Thymosin Beta 4X‐linked expression. Stem Cells Int. 2019;2019:1‐9. 10.1155/2019/9037197 PMC6855023 31781249

[9] Tang H , Chu Y , Huang Z , Cai J , Wang Z . The metastatic phenotype shift toward myofibroblast of adipose‐derived mesenchymal stem cells promotes ovarian cancer progression. Carcinogenesis. 2020;41:182‐193. 10.1093/carcin/bgz083 31046126

[10] Touboul C , Vidal F , Pasquier J , Lis R , Rafii A . Role of mesenchymal cells in the natural history of ovarian cancer: a review. J Transl Med. 2014;12(1):271. 25303976 10.1186/s12967-014-0271-5 PMC4197295

[11] Blake JA , Ziman MR . Pax genes: regulators of lineage specification and progenitor cell maintenance. Development. 2014;141:737‐751. 10.1242/dev.091785 24496612

[12] Epstein JA , Chi N . Getting your Pax straight: Pax proteins in development and disease. Trends Genet. 2002;18(1):41‐47. 11750700 10.1016/s0168-9525(01)02594-x

[13] Shi K , Yin X , Cai MC , et al. PAX8 regulon in human ovarian cancer links lineage dependency with epigenetic vulnerability to HDAC inhibitors. eLife. 2019;8:e44306. 31050342 10.7554/eLife.44306 PMC6533083

[14] Bie LY , Li D , Wei Y , et al. SOX13 dependent PAX8 expression promotes the proliferation of gastric carcinoma cells. Artif Cells Nanomed Biotechnol. 2019;47:3180‐3187. 10.1080/21691401.2019.1646751 31353958

[15] Hardy LR , Pergande MR , Esparza K , et al. Proteomic analysis reveals a role for PAX8 in peritoneal colonization of high grade serous ovarian cancer that can be targeted with micelle encapsulated thiostrepton. Oncogene. 2019;38:6003‐6016. 10.1038/s41388-019-0842-2 31296958 PMC6687548

[16] Ma S , Meng Z , Chen R , Guan KL . The Hippo pathway: biology and pathophysiology. Annu Rev Biochem. 2019;88:577‐604. 10.1146/annurev-biochem-013118-111829 30566373

[17] Rausch V , Hansen CG . The Hippo pathway, YAP/TAZ, and the plasma membrane. Trends Cell Biol. 2020;30:32‐48. 10.1016/j.tcb.2019.10.005 31806419

[18] Koo JH , Guan KL . Interplay between YAP/TAZ and metabolism. Cell Metab. 2018;28:196‐206. 10.1016/j.cmet.2018.07.010 30089241

[19] Kedan A , Verma N , Saroha A , et al. PYK2 negatively regulates the Hippo pathway in TNBC by stabilizing TAZ protein. Cell Death Dis. 2018;9(10):985. 10.1038/s41419-018-1005-z 30250159 PMC6155151

[20] Ferrara AM , De Sanctis L , Rossi G , et al. Mutations in TAZ/WWTR1, a co‐activator of NKX2.1 and PAX8 are not a frequent cause of thyroid dysgenesis. J Endocrinol Invest. 2009;32:238‐241. 19542741 10.1007/BF03346459

[21] Leeman‐Neill RJ , Brenner AV , Little MP , et al. RET/PTC and PAX8/PPARgamma chromosomal rearrangements in post‐Chernobyl thyroid cancer and their association with iodine‐131 radiation dose and other characteristics. Cancer. 2013;119(10):1792‐1799. 10.1002/cncr.27893 23436219 PMC3648615

[22] Di Palma T , Lucci V , de Cristofaro T , Filippone MG , Zannini M . A role for PAX8 in the tumorigenic phenotype of ovarian cancer cells. BMC Cancer. 2014;14:292. 10.1186/1471-2407-14-292 24766781 PMC4005813

[23] Rhodes A , Vallikkannu N , Jayalakshmi P . Expression of WT1 and PAX8 in the epithelial tumours of Malaysian women with ovarian cancer. Br J Biomed Sci. 2017;74:65‐70. 10.1080/09674845.2016.1220709 28367736

[24] Ghannam‐Shahbari D , Jacob E , Kakun RR , et al. PAX8 activates a p53–p21‐dependent pro‐proliferative effect in high grade serous ovarian carcinoma. Oncogene. 2018;37:2213‐2224. 10.1038/s41388-017-0040-z 29379162

[25] Chai HJ , Ren Q , Fan Q , et al. PAX8 is a potential marker for the diagnosis of primary epithelial ovarian cancer. Oncol Lett. 2017;14:5871‐5875. 10.3892/ol.2017.6949 29113220 PMC5661437

[26] Zhou X , Lei QY . Regulation of TAZ in cancer. Protein Cell. 2016;7:548‐561. 10.1007/s13238-016-0288-z 27412635 PMC4980330

[27] Li CG , Nyman JE , Braithwaite AW , Eccles MR . PAX8 promotes tumor cell growth by transcriptionally regulating E2F1 and stabilizing RB protein. Oncogene. 2011;30:4824‐4834. 10.1038/onc.2011.190 21602887 PMC3229668

[28] Hanahan D , Weinberg RA . Hallmarks of cancer: the next generation. Cell. 2011;144:646‐674. 10.1016/j.cell.2011.02.013 21376230

[29] Deng T , Lyon CJ , Bergin S , Caligiuri MA , Hsueh WA . Obesity, inflammation, and cancer. Annu Rev Pathol. 2016;11:421‐449. 10.1146/annurev-pathol-012615-044359 27193454

[30] Chen F , Zhuang X , Lin L , et al. New horizons in tumor microenvironment biology: challenges and opportunities. BMC Med. 2015;13:45. 10.1186/s12916-015-0278-7 25857315 PMC4350882

[31] Grivennikov SI , Greten FR , Karin M . Immunity, inflammation, and cancer. Cell. 2010;140:883‐899. 10.1016/j.cell.2010.01.025 20303878 PMC2866629

[32] Kanai F , Marignani PA , Sarbassova D , et al. TAZ: a novel transcriptional co‐activator regulated by interactions with 14‐3‐3 and PDZ domain proteins. EMBO J. 2000;19:6778‐6791. 11118213 10.1093/emboj/19.24.6778 PMC305881

[33] Zanconato F , Cordenonsi M , Piccolo S . YAP/TAZ at the roots of cancer. Cancer Cell. 2016;29:783‐803. 10.1016/j.ccell.2016.05.005 27300434 PMC6186419

[34] Pobbati AV , Hong W . A combat with the YAP/TAZ‐TEAD oncoproteins for cancer therapy. Theranostics. 2020;10:3622‐3635. 10.7150/thno.40889 32206112 PMC7069086

[35] Pocaterra A , Romani P , Dupont S . YAP/TAZ functions and their regulation at a glance. J Cell Sci. 2020;133(2):jcs230425. 10.1242/jcs.230425 31996398

[36] Horie M , Saito A , Ohshima M , Suzuki HI , Nagase T . YAP and TAZ modulate cell phenotype in a subset of small cell lung cancer. Cancer Sci. 2016;107:1755‐1766. 10.1111/cas.13078 27627196 PMC5198951

[37] Kofler M , Speight P , Little D , et al. Mediated nuclear import and export of TAZ and the underlying molecular requirements. Nat Commun. 2018;9:4966. 10.1038/s41467-018-07450-0 30470756 PMC6251892

[38] Lin KC , Park HW , Guan KL . Regulation of the Hippo pathway transcription factor TEAD. Trends Biochem Sci. 2017;42:862‐872. 10.1016/j.tibs.2017.09.003 28964625 PMC5735856

[39] Nguyen CDK , Yi C . YAP/TAZ signaling and resistance to cancer therapy. Trends Cancer. 2019;5:283‐296. 10.1016/j.trecan.2019.02.010 31174841 PMC6557283

[40] Juan W , Hong W . Targeting the Hippo signaling pathway for tissue regeneration and cancer therapy. Genes. 2016;7(9):55. 10.3390/genes7090055 PMC5042386 27589805

